# Nonmotor Symptoms of Parkinson's Disease

**DOI:** 10.1155/2017/4382518

**Published:** 2017-03-02

**Authors:** Shey-Lin Wu, Rajka M. Liscic, SangYun Kim, Sandro Sorbi, Yuan-Han Yang

**Affiliations:** ^1^Department of Neurology, Changhua Christian Hospital, Changhua, Taiwan; ^2^Department of Neurology, RHON Kliniken, Lehrkrankenhaus Philipps Universität, Marburg, Germany; ^3^Department of Anatomy and Neuroscience, School of Medicine, University of Osijek, Osijek, Croatia; ^4^Neurocognitive Behavior Center, Seoul National University Bundang Hospital, Seongnam, Republic of Korea; ^5^Department of Neuroscience, Psychology, Drug Research and Child Health, University of Florence and IRCCS Don Gnocchi Firenze, Florence, Italy; ^6^Department of Neurology, Kaohsiung Municipal Ta-Tung Hospital, Kaohsiung Medical University, Kaohsiung, Taiwan; ^7^Department of Master's Program in Neurology, Faculty of Medicine, College of Medicine, Kaohsiung Medical University, Kaohsiung, Taiwan; ^8^Department of Neurology, Kaohsiung Medical University Hospital, Kaohsiung Medical University, Kaohsiung, Taiwan

The classical clinical features of the Parkinson's disease (PD) are the motor disorders, in which parkinsonism is defined by the presence of bradykinesia plus at least one additional motor sign, rest tremor, rigidity, or impaired postural reflexes, the well-known clinical criteria [1]. However in the recent decades, scientists and physicians have received a lot of attention of the relevance and frequency of nonmotor symptoms (NMS) independently or dependently along with the motor symptoms [2, 3]. In PD, in general, in its different stages of disease it could be found that overall 98.6% of the PD patients have reported the presence of one or several NMS [4]. Those reported NMS might include olfactory dysfunction, neuropsychiatric manifestations as depression or cognitive impairments, sleep disorders as rapid eye movement behavior disorder, autonomic dysfunctions as gastrointestinal disorders, postural hypotension or urinary disorders, and fatigue pain.

Among NMS, cognitive impairment is one of the most common and significant aspects of PD. The cognitive deficits such as executive deficit or visuospatial disturbances could seriously affect the quality of life, reduce life expectancy, prolong the duration of hospitalization, or therefore increase burdens of caregiver [5, 6]. The pathophysiology of cognitive deficits in PD is complex perhaps due to its complexity and variability from patient to patient. Furthermore, the treatment of cognitive impairment including pharmacotherapy and nonpharmacotherapy (e.g., cognitive training) is still with the limited evidence [7, 8].

Gastrointestinal dysfunction might occur at all stages of PD, often preceding the onset of central motor symptom. Evidence for abnormal *α*-synuclein throughout the enteric nervous system is growing [9]. Different gastrointestinal symptoms, such as dental problem, drooling, dysphagia, gastroparesis, gastroesophageal reflux, constipation, difficult defecation, or loss of weight, are frequent events in all the stages of Parkinson's disease. The treatment of these symptoms is still variable and inconclusive.

In addition to pharmacotherapy, deep brain stimulation (DBS) is a powerful surgical treatment for many aspects of Parkinson disease but lacks consensus inasmuch as the impact of the DBS procedure on executive brain functions [10].

People with PD may experience felt stigma, such as shame, embarrassment, and disgrace, and enacted stigma when encountering responses of others, such as staring, questioning, and avoiding, to their visible features of movement and communication difficulties [11]. The qualitative research may allow a better understanding of a subjective symptom such as stigma in parkinsonian patients from an intercultural and a social point of view.

In order to reach such purposes, this special issue will mainly focus on nonmotor symptoms of PD with its content including above-mentioned topics. We sincerely hope that this special issue will provide interesting new data as well as comprehensive up-to-date reviews for all readers.

*Shey-Lin Wu* *Rajka M. Liscic* *SangYun Kim* *Sandro Sorbi* *Yuan-Han Yang*
